# Differences in Gene Expression and Cytokine Release Profiles Highlight the Heterogeneity of Distinct Subsets of Adipose Tissue-Derived Stem Cells in the Subcutaneous and Visceral Adipose Tissue in Humans

**DOI:** 10.1371/journal.pone.0057892

**Published:** 2013-03-05

**Authors:** Sebastio Perrini, Romina Ficarella, Ernesto Picardi, Angelo Cignarelli, Maria Barbaro, Pasquale Nigro, Alessandro Peschechera, Orazio Palumbo, Massimo Carella, Michele De Fazio, Annalisa Natalicchio, Luigi Laviola, Graziano Pesole, Francesco Giorgino

**Affiliations:** 1 Department of Emergency and Organ Transplantation, Section on Internal Medicine, Endocrinology, Andrology and Metabolic Diseases, University of Bari Aldo Moro, Bari, Italy; 2 Section on General Surgery and Liver Transplantation, University of Bari Aldo Moro, Bari, Italy; 3 Department of Biosciences, Biotechnology and Pharmacological Sciences, University of Bari Aldo Moro, Bari, Italy; 4 Institute of Biomembranes and Bioenergetics, National Research Council, Bari, Italy; 5 Istituto di Ricovero e Cura a Carattere Scientifico Casa Sollievo della Sofferenza, San Giovanni Rotondo (FG), Italy; Children’s Hospital Boston, United States of America

## Abstract

Differences in the inherent properties of adipose tissue-derived stem cells (ASC) may contribute to the biological specificity of the subcutaneous (Sc) and visceral (V) adipose tissue depots. In this study, three distinct subpopulations of ASC, i.e. ASC_SVF_, ASC_Bottom_, and ASC_Ceiling_, were isolated from Sc and V fat biopsies of non-obese subjects, and their gene expression and functional characteristics were investigated. Genome-wide mRNA expression profiles of ASC_SVF_, ASC_Bottom_ and ASC_Ceiling_ from Sc fat were significantly different as compared to their homologous subsets of V-ASCs. Furthermore, ASC_SVF_, ASC_Ceiling_ and ASC_Bottom_ from the same fat depot were also distinct from each other. In this respect, both principal component analysis and hierarchical clusters analysis showed that ASC_Ceiling_ and ASC_SVF_ shared a similar pattern of closely related genes, which was highly different when compared to that of ASC_Bottom_. However, larger variations in gene expression were found in inter-depot than in intra-depot comparisons. The analysis of connectivity of genes differently expressed in each ASC subset demonstrated that, although there was some overlap, there was also a clear distinction between each Sc-ASC and their corresponding V-ASC subsets, and among ASC_SVF_, ASC_Bottom_, and ASC_Ceiling_ of Sc or V fat depots in regard to networks associated with regulation of cell cycle, cell organization and development, inflammation and metabolic responses. Finally, the release of several cytokines and growth factors in the ASC cultured medium also showed both inter- and intra-depot differences. Thus, ASC_Ceiling_ and ASC_Bottom_ can be identified as two genetically and functionally heterogeneous ASC populations in addition to the ASC_SVF_, with ASC_Bottom_ showing the highest degree of unmatched gene expression. On the other hand, inter-depot seem to prevail over intra-depot differences in the ASC gene expression assets and network functions, contributing to the high degree of specificity of Sc and V adipose tissue in humans.

## Introduction

The biological diversity of adipose tissue depots has become a fundamental issue in recent years, in light of its potential impact on human health [1]; [2]. It is known that visceral adipose tissue is morphologically and functionally different from subcutaneous adipose tissue [3]; [4]. Depot-related variations have long been described for a variety of biological endpoints, such as signaling reactions [5], glucose metabolism [6]; [7] and cytokine secretion [8]. It has been also proposed that extrinsic factors, including depot-specific blood flow, cell density, cell heterogeneity and/or innervation [9], could contribute to distinct gene expression patterns and metabolic profiles of adipocytes from different anatomical regions. Alternatively, variations in the inherent properties of undifferentiated fat cell progenitors may dictate the biological specificity between the two fat depots bringing about the innate characteristics of Sc and V adipose cells. This concept has been supported by the demonstration that pre-adipocytes from distinct fat depots retain specific dynamic characteristics and gene expression patterns even after 40 cell doublings [10]. More recently, we have shown that depot-related differences in gene expression, adiponectin secretion, and insulin signaling and action were still evident when precursor cells isolated from bioptic adipose tissue fragments were differentiated in vitro [11]. Altogether, these findings support the concept that there could be an early commitment of fat precursor stromal cells able to influence the biological responses of the resulting adipocytes, independently of extrinsic influences deriving from tissue microenvironment. However, little is known about the identity, localization, or specific characteristics of endogenous adipocyte progenitors.

Adipocyte progenitors recognizably reside in the adipose stromal-vascular fraction (SVF), a heterogeneous mixture of cells operationally defined by enzymatic dissociation of fat tissue followed by density separation from adipocytes [12]. Initially, the stromal-derived cells were designated as «pre-adipocytes», but since 2004 the International Fat Applied Technology Society adopted the term «adipose-derived stem cells» (ASC) to define the plastic-adherent cells isolated from SVF of adipose tissue with self-renewal properties and able to regenerate adipocytes (ASC_SVF_) [12]; [13]. Further complexity to this scenario, however, is provided by the observation that, during the procedure of fat tissue enzymatic digestion and centrifugation, two additional ASC populations can be isolated from the «fat cake» at the top of the supernatant [13]. One ASC population is obtained from a pre-adipocyte fraction in the fat cake not previously collected together with the ASC_SVF_; these cells can be grown at the bottom surface of the culture flask (ASC_Bottom_). Another ASC population develops from mature adipocytes of the fat cake adherent to the ceiling surface of the culture flask filled with growth medium (ASC_Ceiling_). It has been suggested that ASC_Ceiling_ may derive from mature adipocytes through an asymmetric mitosis [14]; [15]. In culture, ASC_Ceiling_ and ASC_Bottom_ display both cell-surface markers that are similar to those expressed by ASC_SVF_, including CD105, SH3, Stro-1, CD49d and CD44, and show similar potential for unlimited self-renewing proliferation and differentiation along the mesenchymal lineage to produce adipocytes, osteoblasts, and chondrocytes [15].

In addition to their ability to differentiate into multiple mesenchymal cell lineages, ASC actively produce paracrine factors, hormones and metabolic signals, in a distinct manner from that of differentiated fat cells. Indeed, non-fat cells and ASC are considered one of the main sources of pro-inflammatory adipokine released by the adipose tissue [16], with great potential for repercussions on distant target tissues and metabolic and cardiovascular regulation [17]. Several studies have reported that ASC and adipocytes contribute roughly similarly to the overall secretion/expression of adipokines, except for adiponectin and leptin [16]; [18]; [19]. Thus, the different production of adipokines, such as IL-6 or MCP-1, between subcutaneous and visceral adipose tissues may reflect either intrinsic properties of the resident ASC or a different ASC proportion within each fat depot [20]; [21], underlying the contribution of ASC to the biological diversity of specific adipose tissue depots.

In this study, ASC_SVF_, ASC_Bottom_ and ASC_Ceiling_ were isolated from abdominal subcutaneous (Sc) and visceral omental (V) adipose tissue biopsies obtained from non-obese subjects and studied through a genome-wide differential gene expression analysis followed by an in-depth bioinformatics examination. In addition, 27 cytokines were measured in the culture medium collected from each of the 6 ASC populations. By performing comparative intra-depot (i.e., ASC_SVF_ vs. ASC_Ceiling_ vs. ASC_Bottom_) and inter-depot (i.e., Sc-ASC vs. V-ASC) analyses, differences in transcripts data and cytokines output emerged, which allowed us to identify six ASC subsets in the Sc and V fat depots that differ among them for gene expression profiles e cell functions.

## Materials and Methods

### Materials

All tissue culture reagents were purchased from Life Technologies (Carlsbad, CA, US). Unless otherwise stated, all chemicals used were obtained from Sigma-Aldrich (St. Louis, MO, US). Recombinant human insulin was purchased from Roche Diagnostics (Mannheim, Germany). The thiazolinedione (TZD) compound rosiglitazone was kindly provided by GlaxoSmithKlein (Middlesex, UK). Recombinant human fibroblast growth factor 2 (FGF2) and EGF were obtained from Miltenyi Biotec (Bergisch Gladbach, Germany).

### Human Donors and Adipose Tissue Biopsies

Paired Sc and V adipose tissue biopsies were obtained from 15 non-obese subjects undergoing elective open-abdominal surgery (9 men, 6 women; age 68±7 yrs; BMI 27.0±1.5 kg/m^2^; fasting plasma glucose 85±11 mg/dl). None of the patients had diabetes or severe systemic illness, and none were taking medications known to affect adipose tissue mass or metabolism. The protocol was approved by the Independent Ethical Committee at the Azienda Ospedaliero-Universitaria Policlinico Consorziale, Bari, Italy. All patients gave their written informed consent.

### ASC Isolation

Human ASC were isolated as previously described [11]; [14], with minor modifications. Briefly, fat tissue fragments were minced and digested in medium containing 1 mg/ml collagenase, type I, with gentle shaking at 37°C for 1 h. Resulting material was filtered through 250 µm mesh, and adipocytes and free oil were separated from stromovascular components by centrifugation at 1,200 rpm for 5 min at room temperature. The floating fraction consisting of pure isolated adipocytes was placed in 25-cm^2^ culture flasks completely filled with DMEM/Ham’s F12 1:1 supplemented with 20% fetal bovine serum, and cells were incubated at 37°C in 5% CO_2_. The primary ASC grown at top and bottom of flask, respectively, were cultured for 7 days until they reached confluence (defined as passage 0), and were then split into 60-mm plates. The stromovascular pellet was resuspended in erythrocyte lysis buffer, consisting of 154 mM NH_4_Cl, 10 mM KHCO_3_, and 0.1 mM EDTA, for 5 min. The cell suspension was centrifuged at 1,200 rpm for 5 min and then resuspended in a culture medium consisting of DMEM/Ham’s F12, 10% FCS and antibiotics. This cell suspension was filtered through a 25-mm sterile nylon mesh before being plated. After a 16-h incubation for cell attachment, cells were cultured in ASC growth medium (DMEM/Ham’s F12 1:1 supplemented with 100 units/ml penicillin, 0.1 mg/ml streptomycin, 2.5% FCS, 1 ng/ml FGF2, and 10 ng/ml EGF). Except when indicated, all cells were used in the experimental procedures at passage 4.

### Mesenchymal Differentiation

For adipogenic differentiation, confluent ASC at passage 4 were differentiated in a chemically defined serum-free medium containing antibiotics, 2 nM triiodotyronine (T3), 100 nM human insulin, 100 nM dexamethasone, and 1 µM rosiglitazone, as previously reported [11]; for the first 4 days of the differentiation period, 0.5 mM of methyl-isobutylxanthine was also added. Osteogenic differentiation was induced as previously described [22]. Differentiation into chondrocytes was induced by StemPro Chondrogenesis Differentiation Medium, according to the manufacturer’s instructions.

### Histochemical Staining of Differentiated Cells

Oil-Red-O, Alizarin Red, and Alcian Blue staining, respectively, were performed as described previously [11]; [22]; [23], and pictures were taken on wide-field microscopes (Nikon) with a color CCD camera.

### RNA Isolation and Quantitative RT-PCR (qRT-PCR)

RNA from the ASC populations was isolated by using an RNeasy kit (Qiagen, Hamburg, Germany). 250 ng of RNA were reverse-transcribed with standard reagents (Applied Biosystems). One microliter of each reverse-transcription reaction was amplified by using SYBR Green PCR master mix from Applied Biosystems, using the ABI 7500 real-time PCR system. For each gene, mRNA expression was calculated relative to 18S rRNA. Amplification of specific transcripts was confirmed by melting curve profiles at the end of each PCR. Primer sequences for each gene are given in Table S1.

### Microarray Analysis

Total RNA was isolated from the ASC of 5 lean subjects. The quality and integrity of total RNA (RNA Integrity Number [RIN] ≥8.0) was evaluated on an Agilent Bioanalyzer (Agilent Technologies, Waldbronn, Germany). RNA was then processed for hybridization on Human Gene 1.0 ST Array chip (Affymetrix, High Wycombe, UK), covering 28,869 well-annotated genes with 764,885 distinct probes, using standard Affymetrix protocols. Raw signal intensities of gene expression data were processed, analyzed, and linearly scaled using GeneChip Operating Software 1.1 (GCOS, Affymetrix) to a mean hybridization intensity of 500 units. For each array, GCOS output was imported as CEL files into Partek Genomic Suite (Partek GS, Partek Inc., 2008, Revision 6.3) software, and data were normalized using the RMA (Robust Multichip Averaging) algorithm. RNA hybridization intensity data obtained from the array analysis were concordant for all ASC_Bottom_, and for 4 of the 5 ASC_Ceiling_ and ASC_SVF_ from both Sc and V fat depot. The remaining one ASC samples were excluded from the array analysis, since their data were ambiguous or incorrect, resulting in cell-mismatched design when compared with the homologous Sc and V-ASC subsets. Similarities and differences among gene expression profiles were assessed by hierarchical clustering using Partek GS software. Statistical significance was defined as being differentially expressed with an adjusted *p*-value <0.01. The array data have been deposited at the Gene Expression Omnibus archive, accession number GSE37324. A further analysis to identify the biological mechanisms, pathways and functions and the most relevant overexpressed genes was performed using the Ingenuity Pathways Analysis (IPA) software and database (Ingenuity Systems, http://www.ingenuity.com). Briefly, the dataset containing gene identifiers and corresponding fold changes was uploaded into the web-delivered application, and each gene identifier was mapped to its corresponding gene object. The threshold for a significant association was determined by the *p*-value <0.01, becoming significant any score above 3 [24]; [25]. For all analyses, Fisher’s exact test was used to calculate a *p*-value and to determine a score for all networks that were ranked on the probability that a collection of genes equal to or greater than the number in a network could be achieved by chance alone.

### Multiplex Analysis of Cytokine Production

ASC-derived conditioned medium (CM) was obtained by incubating confluent cells in serum-free DMEM/Ham’s F12 for 16 h, after which time all CM was collected, pooled, cleared by centrifugation, frozen in dry ice, and stored at −80°C. Concentrations of various cytokines in CM were analyzed using a multiplex biometrix immunoassay from Bio-Rad, containing fluorescent dyed microspheres conjugated with a monoclonal antibody specific for the target proteins according to the manufacturer’s instructions (Bio-Plex Human Cytokine Assay; Bio-Rad, Munich, Germany). The following cytokines were assayed: IL-1β, IL-1ra, IL-2, IL-4, IL-5, IL-6, IL-7, IL-8, IL-9, IL-10, IL-12 (p70), IL-13, IL-15, IL-17, FGF basic, eotaxin, G-CSF, GM-CSF, IFN-γ, IP-10, MCP-1 (MCAF), MIP-1α, MIP-1β, PDGF-BB, RANTES, TNF-α, and VEGF. The amount of protein in the medium was normalized for 10^6^ cells [26]. The inter- and intra-assay coefficients of variation for all cytokines under investigation were less than 10%.

### Statistical Analysis

Data are presented as mean ± SE. Comparisons between the values were performed using a two-tailed Student’s t-test. For the comparison of multiple groups, a one-way ANOVA test followed by Fisher’s post-hoc test was applied. For all statistical analyses, the level of significance was set at a probability of *p*<0.05. All experiments were repeated at least 3 times. These analyses were performed using SPSS 16 (SPSS, Chicago, IL).

## Results

### Phenotypic Analysis and Stem Cell Properties of ASC Populations

**Figure 1:**
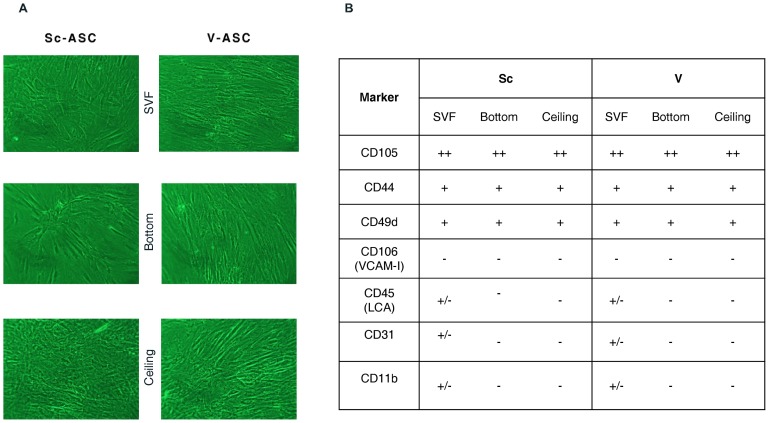
Isolation and characterization of human ASC. *A*. Morphology of human ASC populations, as detected under bright field. *B*. Expression of specific markers measured by *q*RT-PCR. Cells were harvested at passage 0, and RNA was analyzed for markers of adipose-derived stem cells (CD105, CD44, CD49d), human leukocytes and macrophages (CD45, CD11b), and mature endothelial cells (CD31).+or – signs indicate the relative levels of marker expression. Results are from 5 independent adipose tissue donors.

**Figure 2:**
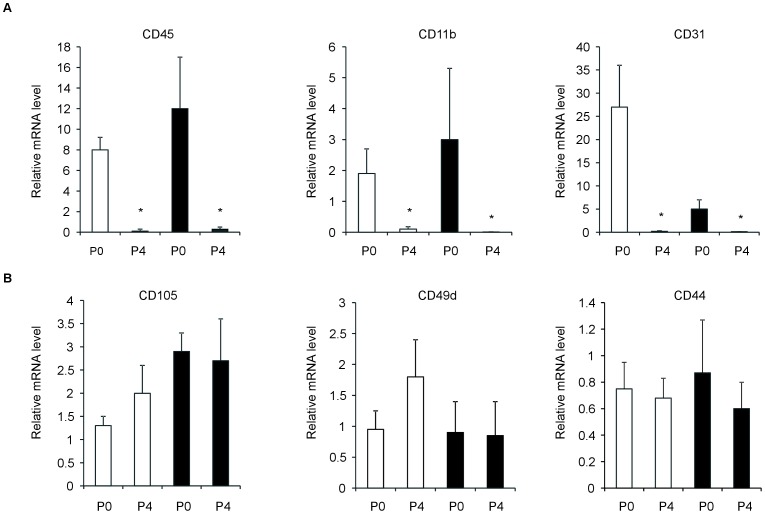
mRNA expression levels of contaminating mesenchymal lineage markers (CD45, CD31, CD11b) (*A*) and adipose-derived stem cell markers (CD105, CD44, CD49d) (*B*), measured by *q*RT-PCR. ASC_SVF_ were analyzed at passage 0 (P0) and at passage 4 (P4). Open bars, Sc-ASC; filled bars, V-ASC. *p<0.001 vs. P0. Results are from 5 independent adipose tissue donors.

ASC_SVF_, ASC_Ceiling_ and ASC_Bottom_ isolated from the Sc and V fat depots grew as a characteristic cell monolayer in culture dishes (Fig. 1*A*). To identify these cells as adipocyte progenitors and to rule out potential contamination by other adipose tissue cell types, analysis by *q*RT-PCR for a selection of known hematopoietic, endothelial and stem cell-associated markers was carried out. The expression of the key ASC markers CD105, CD49d, and CD44 was noticeably pronounced and expressed at similar magnitude in ASC_SVF_, ASC_Ceiling_ and ASC_Bottom_ from both Sc and V fat depots (Fig. 1*B*; Table S2). The initial Sc-ASC_SVF_ and V-ASC_SVF_ cultures contained a subset of cells that were also positive for the endothelial and hematopoietic lineage markers CD11b, CD45, and CD31 (Fig. 2*A*). However, with successive passages, the expression of CD11b, CD45, and CD31 declined significantly, becoming negligible by passage 4 (*p*<0.001 vs. passage 0; Fig. 2*A*), whereas expression levels of ASC markers remained constant (*p* = 0.860 vs. passage 0; Fig. 2*B*).

To confirm the multipotent mesenchymal stem cell properties of the distinct ASC populations, a comparative in vitro tri-lineage differentiation assay was carried out by exposing culture-expanded ASC derived from Sc and V adipose tissue to osteogenic, chondrogenic, or adipogenic differentiation media, respectively. The qualitative histochemical evaluation of functional outcomes indicated that ASC_SVF_, ASC_Ceiling_ and ASC_Bottom_ from both adipose tissue depots underwent differentiation along the adipogenic (assayed by Oil-Red-staining), osteogenic (assayed by Alizarin Red staining) and chondrogenic (assayed by alcian blue staining of sections derived from 2-week-old pellet cultures) lineages, respectively (Fig. S1). The mRNA levels of fat cell-specific gene markers, including GLUT4, PPARγ2 and adiponectin, were also increased several-fold with adipocyte differentiation (*p*<0.001 vs. ASC), and found to be similarly expressed in the distinct ASC populations (Fig. S2). These results indicate that the differentiation process into mature adipocytes was completed in an apparently equal manner in all ASC populations.

### Gene Expression Profiles of ASC Populations

**Figure 3:**
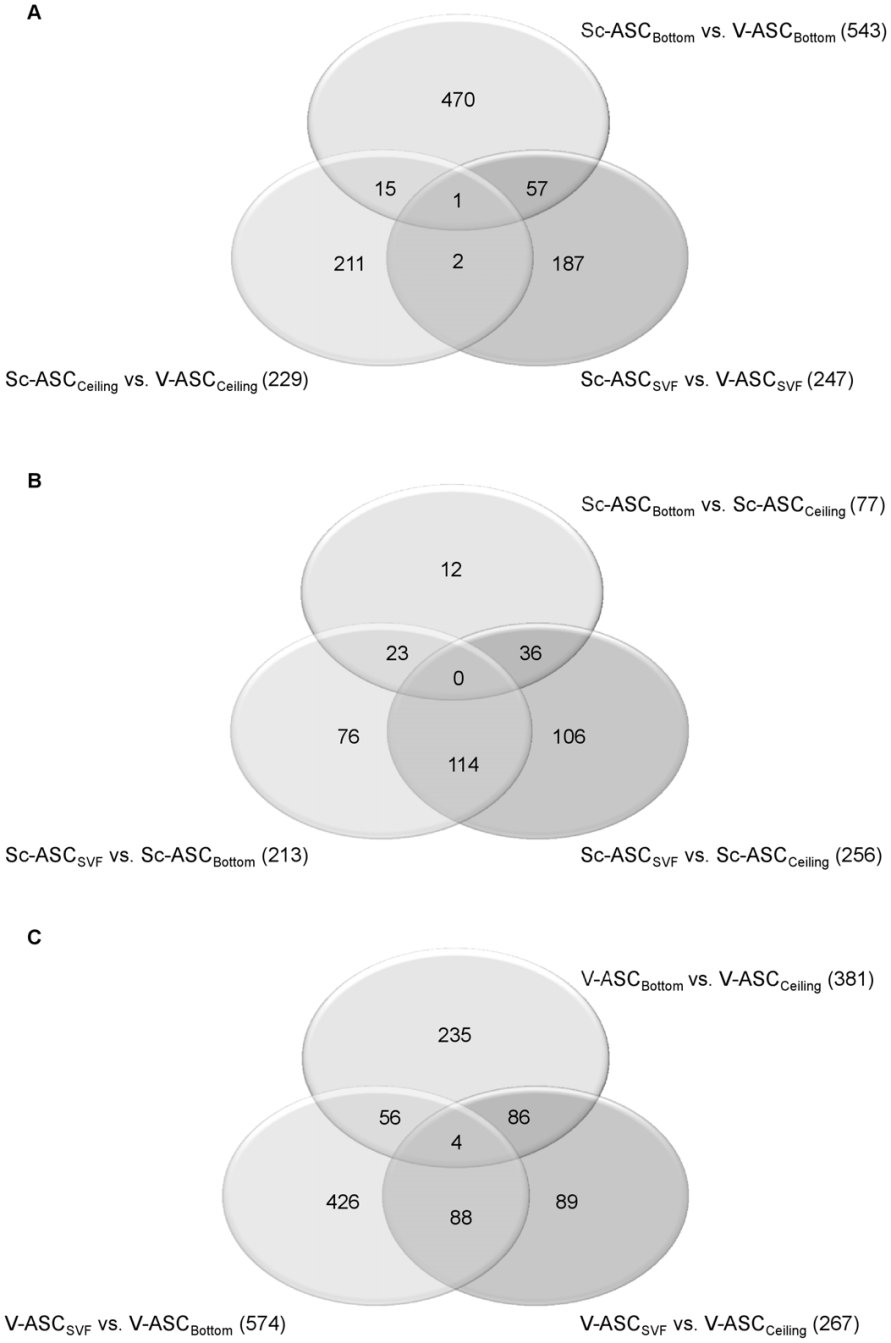
Venn diagrams summarizing the number of differentially expressed genes in Sc and V ASC populations. *A*. Genes found to be differentially expressed by comparing Sc-ASC and V-ASC subsets (inter-depot analysis). *B*. Genes found to be differentially expressed by comparing ASC_SVF_, ASC_Bottom_, and ASC_Ceiling_ from the Sc adipose tissue (Sc intra-depot analysis). *C*. Genes found to be differentially expressed by comparing ASC_SVF_, ASC_Bottom_, and ASC_Ceiling_ from the V adipose tissue (V intra-depot analysis). In each panel, figures for conjoint (and non-conjoint) differentially expressed genes are also indicated.

To investigate the gene expression profile of ASC_SVF_, ASC_Ceiling_ and ASC_Bottom_ isolated from Sc and V fat depots, a genome-wide analysis was performed using an Affimetrix Human Gene 1.0 ST Array chip microarray with 764,885 probe sets representing 28,869 different annotated genes (Fig. S3). Of these, 1,019 genes were identified that differed significantly between Sc-ASC and V-ASC (Fig. 3*A*). These genes were identified by first testing for significant expression differences among all ASC populations using the ANOVA model implemented in Partek GS at a *p*-value <0.05. Then, on genes that showed a statistically significant difference, a secondary filter was applied to select those genes that varied at least 1.5-fold. Thus, of the selected 1,019 genes, 247 genes were found to be differentially expressed between Sc-ASC_SVF_ and V-ASC_SVF_, 543 between Sc-ASC_Bottom_ and V-ASC_Bottom_, and 229 between Sc-ASC_Ceiling_ and V-ASC_Ceiling_ (Fig. 3*A*). Of these 1,019 genes, 75 (7.4%) were found to have a conjoint differential expression in ASC_SVF_, ASC_Ceiling_ and ASC_Bottom_, while 868 (85.2%) showed a differential expression unique to each ASC subset, respectively (Fig. 3*A*). One gene was found to have a conjoint differential expression in all three ASC populations (Fig. 3*A*; Table S3).

A similar analysis was conducted on the ASC_SVF_, ASC_Bottom_ and ASC_Ceiling_ populations within each adipose tissue depot, again selecting those genes that varied significantly and at least 1.5-fold as compared to the baseline gene expression level (Fig. 3*B*). In Sc-ASC populations, 256 genes were found to be differentially expressed between ASC_SVF_ and ASC_Ceiling_, 213 between ASC_SVF_ and ASC_Bottom_, and 77 between ASC_Bottom_ and ASC_Ceiling_ (Fig. 3*B*). Of these 546 genes, 173 (31.7%) were found to have a conjoint differential expression in ASC_SVF_, ASC_Ceiling_ and ASC_Bottom_, while 194 (35.5%) showed a differential expression unique to each ASC subset, respectively (Fig. 3*B*). No gene was found to have a conjoint differential expression in all three Sc-ASC populations (Fig. 3*B*). In V-ASC populations, 267 genes were differentially expressed between ASC_SVF_ and ASC_Ceiling_, 574 between ASC_SVF_ and ASC_Bottom_, and 381 between ASC_Bottom_ and ASC_Ceiling_ (Fig. 3*C*). Of these 1,222 genes, 234 (19.1%) were found to have a conjoint differential expression in ASC_SVF_, ASC_Ceiling_ and ASC_Bottom_, while 750 (61.4%) showed a differential expression unique to each ASC subset, respectively (Fig. 3*C*). In contrast to the Sc adipose tissue, 4 genes were found to have a conjoint differential expression in all three V-ASC populations (Fig. 3*C*; Table S3). Altogether these findings indicate that the distinct three ASC populations show larger variations in gene expression when inter-depot as compared to intra-depot analyses are carried out, and that intra-depot variations in gene expression are apparently smaller for Sc-ASC than for V-ASC subsets.

**Figure 4:**
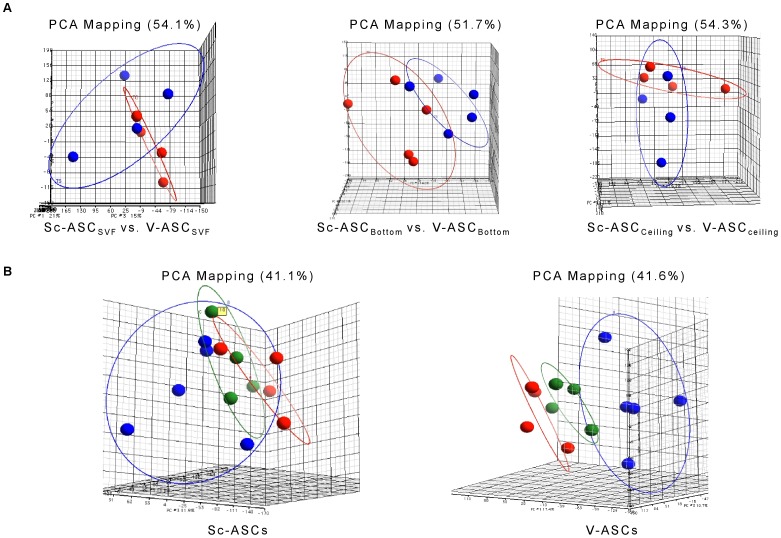
Global views of gene expression utilizing the Principal Component Analysis (PCA). *A*. PCA comparing gene expression of Sc-ASC (blue) and V-ASC (red) subsets. *B*. PCA comparing gene expression of ASC_SVF_ (red), ASC_Bottom_ (blue), and ASC_Ceiling_ (green) from Sc and V fat depots, respectively. PCA analyses were performed by Partek GS software using default settings that include a threshold to remove low background level intensities. PCA percent mapping on the top of each plot indicates the explained variability on the first coordinate.

To analyze inter- and intra-depot differences in gene expression profiles further, the principal component analysis (PCA) and hierarchical clusters analysis were applied. PCA was used to demonstrate similarities and differences in the transcriptional profiles of the analyzed samples. On a PCA plot, cell types with similar expression profiles can be positioned in proximity to each other. The position of each cell sample was plotted against the PC1, PC2, and PC3 axes in a three-dimensional (3D) space (Fig. 4*A*). When ASC_SVF_, ASC_Bottom_, and ASC_Ceiling_ from the Sc adipose tissue depot were compared with the corresponding ASC from V fat, the PCA delineated two distinct positions in the 3D space for each ASC subset (Fig. 4*A*). In addition, when comparisons were carried out within each fat depot, the PCA depicted three clearly separated groups, consisting of ASC_SVF_, ASC_Ceiling_ and ASC_Bottom_, showing the existence of ASC populations that are clearly heterogeneous in their gene expression in both the Sc and V adipose tissues (Fig. 4*B*).

However, a high degree of heterogeneity was found for ASC_Bottom_, whereas ASC_SVF_ and ASC_Ceiling_ were found to be yet distinct but more closely related (Fig. 4*B*). Gene expression profiles were then subjected to hierarchical clusters analysis. A more striking difference was found by comparing each ASC subset from Sc and V adipose tissue than ASC subsets within Sc or V adipose tissue depots (Fig. S4 *A*). However, in both depots ASC_SVF_ and ASC_Ceiling_ displayed a rather related gene expression pattern over multiple conditions, whereas ASC_Bottom_ showed a highly dissimilar pattern compared to ASC_SVF_ and ASC_Ceiling_ (Fig. S4 *A*).

### Functional Network Analysis of Differentially Expressed Genes in the ASC Subsets

To provide an insight into the potential functional implications of the observed differences in gene expression, the transcripts whose expression varied at least 1.5-fold and that were ordered identically along both axes by hierarchical cluster analysis were placed in the context of the known interactome using the Ingenuity Pathway Analysis (IPA). The score was then determined for each network based on *p*-value. Scores of 3 or higher were considered significant with 99% confidence. Using these criteria and gene ontology analysis, five different highly significant networks with a score of at least 20 were identified (Table 1). As expected for data derived from adipose precursor cells, the top functions of the highly significant networks were associated with lipid and carbohydrate metabolism, cell morphology, cell cycle, cellular assembly and organization, cellular movement and embryonic and tissue development (Table 1). Although there was some overlap in functions, there was also a clear distinction between each Sc-ASC and their corresponding V-ASC subsets, and among ASC_SVF_, ASC_Bottom_, and ASC_Ceiling_ of Sc or V fat depots (Table 1).

**Table 1:** Significant top list of associated network functions from IPA in Sc and V ASCs.

ID	Associated Network Fuctions	Score	
1	Carbohydrate Metabolism. Lipid Metabolism. Small Molecule Biochemistry	42	Sc-ASC_SVF_
2	DNA Replication. Recombination. and Repair. Post-Traslational Modification. Protein Folding	42	
3	Genetic Disorder. Lipid Metabolism. Molecular Transport	41	
4	Cellular Assembly and Organization. Lipid Metabolism. Molecular Transport	39	
5	Cellular Assembly and Organization.Cardiovascular System Development and Function. Cell Cycle	33	
1	Organismal Injury and Abnormalities. Carbohydrate Metabolism. Lipid Metabolism	48	Sc-ASC_Bottom_
2	Nervous System Development and Function. Cell Signaling. Carbohydrate Metabolism	45	
3	Connective Tissue Disorders. Genetic Disorder. Dermatological Diseases and Conditions	33	
4	Cell Morphology. Cellular Movement. Immune Cell Trafficking	31	
5	RNA Damage and Repair. Gene Expression. RNA Post-Transcriptional Modification	28	
1	Cell Morphology. Cellular Assembly and Organization. Auditory Disease	43	Sc-ASC_Ceiling_
2	Post-Translational Modification. Protein Folding. Genetic Disorder	41	
3	Cellular Function and Maintenance. Cellular Assembly and Organization. Cellular Movement	39	
4	DNA Replication. Recombination. and Repair. Cellular Assembly and Organization. Cellular Function and Maintenance	39	
5	Nervous System Development and Function. Development Disorder. Genetic Disorder	36	
1	Lipid Metabolism. Small Molecule Biochemistry. Cell Morphology	44	V-ASC_SVF_
2	Post-Translation Modification. Protein Folding. Cellular Assembly and Organization	41	
3	DNA Replication. Recombination. and Repair. Carbohydrate Metabolism. Small Molecule Biochemistry	39	
4	Post-Traslation Modification.DNA Replication. Recombination. and Repair. Nucleic Acid Metabolism	30	
5	Cellular Growth and Proliferation. Hematological System Development and Function. Lymphoid Tissue Structure and Development	21	
1	Cellular Function and Maintenance. Embryonic Development. Lipid Metabolism	56	V-ASC_Bottom_
2	Cancer. Hematological Disease. Immunological Disease	39	
3	Amino Acid Metabolism. Protein Synthesis. Small Molecule Biochemistry	24	
4	Cell Cycle. Cell Morphology. Cellular Development	20	
5	Cellular Function and Maintenance. Hematopoiesis. Cell Cycle	20	
1	DNA Replication. Recombination. and Repair. Cellular Movement. Nervous System Development and Function	50	V-ASC_Ceiling_
2	Post-Translational Mopdification. Protein Folding. Molecular Transport	42	
3	Cell Signaling. Cellular Response to Therapeutics. Lipid Metabolism	28	
4	Post-Translational Mopdification. DNA Replication. Recombination. and Repair. Nucleic Acid Metabolism	27	
5	Cellular Development. Cell Cycle. Cellular Assembly and Organization	25	

IPA also performed a canonical pathway analysis in order to associate differentially regulated genes with known specific biological pathways (Table 2). Also in this case, the top ranked upregulated canonical pathways displayed some overlaps, yet distinctions were observed both when each Sc-ASC population was compared with the corresponding V-ASCs and among ASC_SVF_, ASC_Ceiling_ and ASC_Bottom_ from each fat depot. The most significant pathways identified in Sc-ASC included endoplasmic reticulum stress pathway, G-protein coupled receptor signaling, and ATM signaling, while those found in V-ASC included oxidative phosphorylation, protein ubiquitination pathway, and cell cycle: G1/S checkpoint regulation. These results further underline the inter-depot and intra-depot functional heterogeneity of the human ASC.

**Table 2:** Significant top list of canonical pathways from IPA in Sc and V ASCs.

Top Canonical Pathways	*p*-Value	Ratio	
Endoplasmic Reticulum Stress Pathway	3.70E−04	4/18 (0.222)	Sc-ASC_SVF_
ATM Signaling	5.43E−04	6/54 (0.111)	
Protein Ubiquitination Pathway	8.46E−04	14/274 (0.051)	
CD40 Signaling	1.51E−03	6/70 (0.086)	
DNA Double-Strand Break Repair by Non-Homologous End Joining	2.38E−03	3/19 (0.158)	
G-Protein Coupled Receptor Signaling	1.27E−02	17/531 (0.032)	Sc-ASC_Bottom_
Primary Immunodeficiency Signaling	6.68E−02	3/63 (0.048)	
IL-9 Signaling	1.23E−01	2/40 (0.05)	
Basal Cell Carcinoma Signaling	1.33E−01	3/73 (0.041)	
Role of NANOG in Mammalian Embryonic Stem Cell Pluropotency	1.40E−01	4/117 (80.034)	
Endoplasmic Reticulum Stress Pathway	5.93E−04	4/18 (0.222)	Sc-ASC_Ceiling_
NRF2-mediated Oxidative Stress Response	7.67E−04	12/192 (0.062)	
Protein Ubiquitination Pathway	9.81E−04	15/274 (0.055)	
ATM Signaling	1.04E−03	6/54 (0.111)	
CD40 Signaling	2.82E−03	6/70 (0.086)	
Protein Ubiqitination Pathway	8.56E−06	13/274 (0.047)	V-ASC_SVF_
Cell Cycle: G1/S Checkpoint Regulation	3.46E−03	4/61 (0.066)	
Purine Metabolism	3.63E−03	11/439 (0.025)	
Role of CHK Proteins in Cell Cycle Checkpoint Control	5.77E−03	3/35 (0.086)	
DNA Double-Strand Break Repair by Homologous Recombination	1.02E−02	2/17 (0.118)	
Oxidative Phosphorylation	3.44E−06	10/165 (0.061)	V-ASC_Bottom_
Mitochondrial Dysfunction	2.96E−03	6/169 (0.036)	
Ubiquinone Biosynthesis	1.03E−02	4/119 (0.034)	
Agrin interactions at Neuromuscular Junction	2.72E−02	3/69 (0.043)	
Protein Ubiquitination Pathway	3.94E−02	6/274 (0.022)	
Protein Ubiqitination Pathway	6.17E−04	11/274 (0.04)	V-ASC_Ceiling_
Cell Cycle: G1/S Checkpoint Regulation	5.65E−03	4/61 (0.066)	
Chronic Myeloid Leukemia Signaling	8.07E−03	5/108 (0.046)	
Role of CHK Proteins in Cell Cycle Checkpoint Control	8.44E−03	3/35 (0.086)	
Purine Metabolism	9.98E−03	11/439 (0.025)	

### Confirmation of Gene Expression Differences by qRT-PCR

**Figure 5:**
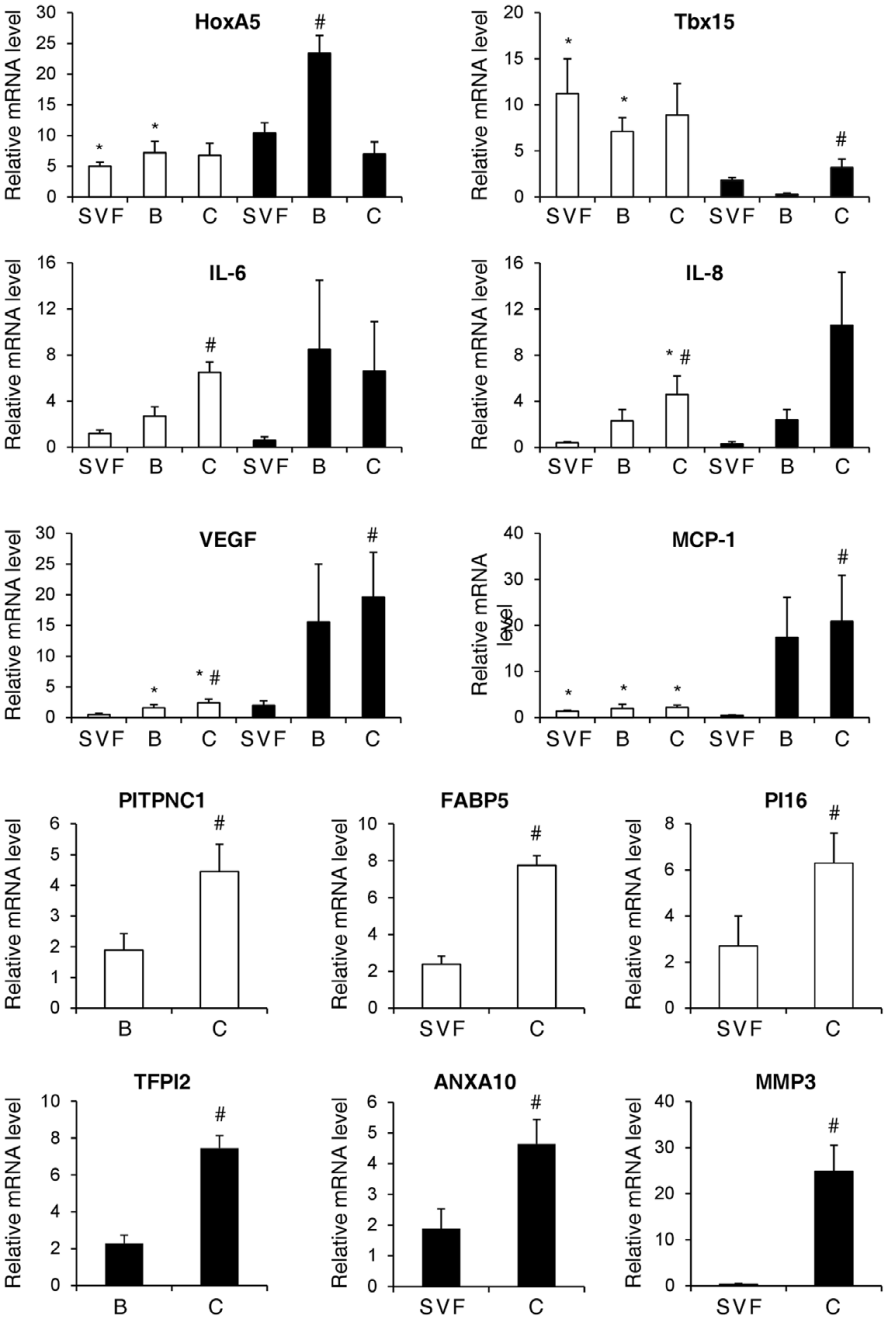
Quantitative analysis of specific genes previously identified in the microarray analysis and found to be differentially expressed in the distinct ASC subsets. mRNA levels were analyzed by *q*RT-PCR, as described under Methods. Open bars, Sc-ASC; filled bars, V-ASC. All data represent mean ± SE from 9 independent adipose tissue donors. *p<0.05 vs. V-ASC; #*p*<0.05 vs. other ASC populations from the same adipose tissue depot (ANOVA test followed by Fisher’s post-hoc test). SVF, stromo-vascular; B, bottom; C, ceiling.

The array analysis described above provided us with trends reflecting changes in the expression of several mRNA species in the distinct ASC subsets. To better appreciate which of these findings were reproducible by standard and more rigorous molecular testing and to what extent such analysis would provide comparable fold change values, specific candidate genes were selected for further study using *q*RT-PCR. Out of the 1,019 differentially expressed genes changing at least 1.5-fold (by Affimetrix analysis) in Sc-ASC compared to V-ASC, 12 genes, known to be involved in the above mentioned top ranked functional networks and canonical pathways, were selected (quantitative data from array analysis are shown in Table S4). These include HoxA5 and Tbx15 (embryonic development), FABP5 (lipid homeostasis), IL-6, IL-8, MCP-1, VEGF, and MMP3 (immune system and proinflammatory signal transduction), PI16, PITPNC1, TFPI2 and ANXA10 (cell cycle). As shown in Figure 5, all of these candidate genes were also found to be differently expressed by *q*RT-PCR analysis. Specifically, HoxA5 mRNA levels were more expressed in V-ASC_SVF_ and V-ASC_Bottom_ compared to Sc-ASC_SVF_ and Sc-ASC_Bottom_, respectively (*p*<0.01), whereas Tbx15 mRNA expression was higher in Sc- than V-ASC (*p*<0.01). IL-8 mRNA was expressed at higher levels in V-ASC_Ceiling_ than in Sc-ASC_Ceiling_, and VEGF and MCP-1 mRNA levels were higher in V-ASC_Bottom_ and V-ASC_Ceiling_ than in Sc-ASC_Bottom_ and Sc-ASC_Ceiling_, respectively (*p*<0.01). Intra-depot differences in mRNA levels of specific genes were also found. For example, HoxA5 mRNA levels were 2.3-fold higher in V-ASC_Bottom_ compared to V-ASC_SVF_ and V-ASC_Ceiling_, respectively (Fig. 5; *p*<0.01). Tbx15, VEGF and MCP-1 mRNA levels were higher in V-ASC_Ceiling_ than in V-ASC_SVF_ (Fig. 5; *p*<0.05). IL-8, IL-6 and VEGF mRNA levels were significantly higher in Sc-ASC_Ceiling_ compared to Sc-ASC_SVF_ and Sc-ASC_Bottom_ (Fig. 5; *p*<0.05). mRNA levels of PITPNC1, FABP5 and PI16 were higher in Sc-ASC_Ceiling_ than in Sc-ASC_SVF_ or Sc-ASC_Bottom_, and TFPI2, ANXA10 and MMP3 were expressed at higher levels in V-ASC_Ceiling_ than in V-ASC_SVF_ or V-ASC_Bottom_, as detailed in Fig. 5 (*p*<0.02).

### Endocrine Activity of ASC Populations

**Figure 6:**
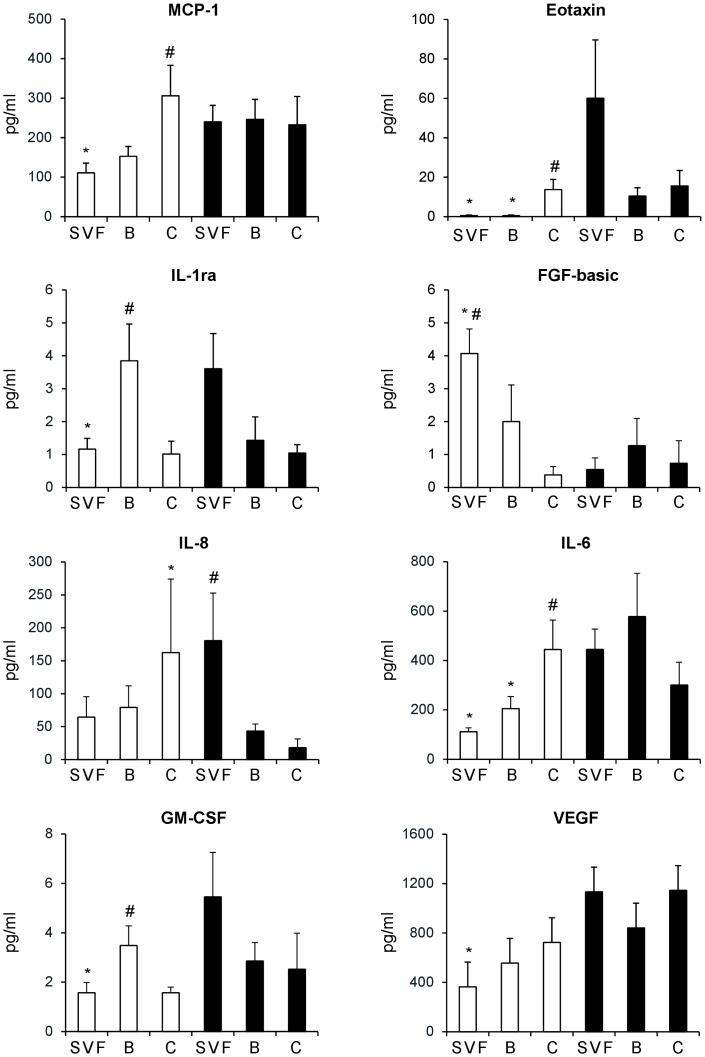
Release of cytokines from Sc-ASC and V-ASC populations. Culture medium from Sc-ASC (open bars) and V-ASC (filled bars) subsets (10^6^ cells) was collected after a 16-h period, and levels of specific cytokines were determined using the multiplex technique, as described under Materials and Methods. Data represent the mean ± SE of results from 9 independent adipose tissue donors. *p<0.05 vs. V; #*p*<0.05 vs. other ASC subsets from the same adipose tissue depot (ANOVA test followed by Fisher’s post-hoc test).

To complement the findings on the differences in gene expression patterns of the distinct ASC populations with information on their endocrine activity, the release of cytokines, chemokines, and growth factors in the conditioned medium (CM) was also measured using a multiplex detection system capable of analyzing 27 different protein factors. Of the 27 molecules specified in the array, 14 (i.e., IL-2, IL-4, IL-5 IL-7, IL-9, IL-10, IL-12, IL-13, MIP1-α, MIP1-β, PDGF-BB, FGF-basic, GM-CSF, IP-10) were found to be not released at significant levels into the CM collected after 16 h. In contrast, 13 molecules were detectable (i.e., IL-1β, IL-1ra, IL-15, IL-17, G-CSF, IFN-γ, RANTES, TNF-α, eotaxin, IL-8, MCP-1, VEGF, IL-6), and, of these, MCP-1, Eotaxin, IL-1ra, FGF-basic, IL-6, IL-8, GM-CSF, and VEGF were above 50% of the positive control in all examined samples and also significantly different in the distinct ASC subsets of both Sc and V adipose tissue. Specifically, protein levels of MCP-1, eotaxin, IL-1ra, IL-6, GM-CSF, and VEGF were significantly higher in V-ASC_SVF_ compared to Sc-ASC_SVF_ CM (Fig. 6; *p*<0.05). In addition, the protein levels of eotaxin and IL-6 were also higher in V-ASC_Bottom_ than in Sc-ASC_Bottom_ CM (Fig. 6; *p*<0.03). In contrast, secretion of IL-8 was significantly increased in Sc-ASC_Ceiling_ compared to V-ASC_Ceiling_ CM (Fig. 6; *p*<0.01). MCP-1, eotaxin, and IL-6 were secreted at higher levels in Sc-ASC_Ceiling_ than in Sc-ASC_SVF_ and Sc-ASC_Bottom_ CM (Fig. 6; *p*<0.05), whereas release of IL-1ra and GM-CSF was significantly greater in Sc-ASC_Bottom_ compared to Sc-ASC_SVF_ and Sc-ASC_Ceiling_ CM (Fig. 6; *p*<0.05). Finally, IL-8 was significantly increased in V-ASC_SVF_ as compared to V-ASC_Bottom_ and V-ASC_Ceiling_ CM (Fig. 6; *p*<0.05). These results show that ASC_SVF_, ASC_Ceiling_ and ASC_Bottom_ differ from each other also in terms of their ability to release specific cytokines and growth factors in the culture medium under basal conditions.

## Discussion

The dynamics of adipocytes turnover is considered to play a remarkable role in regulating the total fat mass. Adipocytes are continuously produced in adult human adipose tissue, and even more so in obese individuals [27]. Furthermore, ASC originating from the SVF of adult adipose tissue are capable of reconstituting the adipocyte mass in lipodystrophy syndromes [28]. It should be also emphasized that the ASC component represents 15% to 50% of the cells in adipose tissue and actively produces paracrine factors and hormones in a manner distinct from that of differentiated fat cells [29]. Thus, identifying the source and inherent properties of ASC is key to understand the plasticity of adipose tissue in humans and its relation to obesity and the associated metabolic abnormalities.

Previous studies have analyzed the gene expression profiles of ASC, focusing on i.) gene expression changes during *in vitro* differentiation to osteogenic, chondrogenic or adipogenic lineages [30]; [31], ii.) comparative analysis of bone marrow and adipose tissue-derived mesenchymal stromal cells in *in vitro* adipogenic, chondrogenic or osteogenic differentiation [32]; [33], and iii.) comparative transcriptomics of human multipotent stem cells during adipogenesis and osteoblastogenesis [34]. However, this is the first time that a quantitative large-scale gene expression analysis of ASC_SVF_, ASC_Bottom_, and ASC_Ceiling_ from distinct adipose tissue depots, showing significant intra- and inter-depot heterogeneity of the ASC subsets, has been carried out.

Phenotypically, and in line with previous studies [12]; [14]; [15], the progenitor cells isolated from Sc and V adipose tissue were defined as mesenchymal stem cells (MSC) by a panel of surface markers, subsequent adherence to plastic surface, and their propensity to give rise to adipocytes, chondrocytes, and osteocytes (Figs. 1–2, and Figs. S1, S2, S3). All subsets of ASC isolated from both fat depots fulfilled these characteristics without showing major differences among them, at least when exposed to standard culture and differentiation conditions (Figs. 1–2, and Figs. S1, S2, S3). This indicates that, by using standardized procedures, we were able to reproducibly establish apparently homogenous Sc-ASC and V-ASC populations. However, by using gene arrays analysis, more than 1,000 mRNAs were found to be significantly different and variant more than 1.5-fold between Sc-ASC and V-ASC (Fig. 3). Furthermore, ASC populations (i.e., ASC_SVF_, ASC_Ceiling_ and ASC_Bottom_) originating from the same adipose tissue depot, Sc or V, were also surprisingly distinct from each other for gene expression profiles (Fig. 3). The significant gene expression heterogeneity between Sc-ASC and V-ASC subsets and among intra-depot ASC subsets was confirmed by PCA, an unbiased method to discriminate biological differences [35], which showed clearly separated three-dimensional space projections of the distinct gene expression profiles (Fig. 4, *A* and *B*). The tree-like diagram, which illustrates the hierarchical relationship among genes in a dataset in which the distance from the root to a cluster indicates the similarity of the cluster [35], further sustained the degrees of homogeneity or more distant relationship among groups of closely related genes between Sc-ASC and V-ASC populations and among ASC_SVF_, ASC_Ceiling_ and ASC_Bottom_ from the same fat depot (Fig. S4, *A* and *B*). The dendrogram in Figure S4 corroborates a far more striking difference between each Sc-ASC_SVF_, Sc-ASC_Ceiling_ and Sc-ASC_Bottom_ and its own homologous V-ASC subset. Taking into account that ASC_Ceiling_ have been suggested to develop from an “asymmetric mitosis” of terminally differentiated adipocytes [14]; [15], it is quite surprising to find that ASC_Ceiling_ and ASC_SVF_ shared a similar degree of closely related genes, which conversely was highly different when compared to the gene expression pattern of ASC_Bottom_ (Fig. S4 *B*). After considering that ASC_Bottom_ derive from precursors present in the fat cake at the top of the supernatant (Fig. S3), and thus could be considered as tissue-resident adipose stem cells, it follows that these results establish, for the first time, the existence of two different tissue-resident adipose stem cells, i.e., ASC_Bottom_ and ASC_SVF_, characterized by an unmatched gene expression pattern within the same fat depot. In addition, the comparison of different ASC subsets by hierarchical cluster analysis demonstrated a relatively close resemblance of the profiles among all five donor samples for each subset (Fig. S4), indicating a relatively low inter-individual variation in ASC gene expression patterns.

Investigation of how the genes differently expressed by ASC_SVF_, ASC_Bottom_, and ASC_Ceiling_ might interact as part of complex pathways and network functions by using the Ingenuity Pathway Analysis made it possible to reveal several and distinct functional networks between Sc-ASC and V-ASC, and among ASC_SVF_, ASC_Bottom_ and ASC_Ceiling_ (Tables 1 and 2). A simultaneous survey and evaluation of the top canonical pathways and associated network functions displayed that lipid and carbohydrate metabolism, cell morphology, cell cycle, cellular assembly and organization, cellular movement and embryonic and tissue development showed highly significant differences in ASC populations (Tables 1 and 2). In addition, it should be emphasized that such analyses reflect relationships of currently known genes and participation in established pathways and do not consider the potential contribution of novel genes and functions that are yet to be identified.

Confirmation of differences in network functions was also achieved by evaluating the mRNA expression of 12 genes that varied at least 1.5-fold from baseline gene expression, and are known to be involved in the above networks and up-regulated canonical pathways (Fig. 5). Interestingly, as previously observed in other studies on fat precursors and mature adipocytes, HoxA5, VEGF, IL-8 and MCP-1 mRNAs were significantly more expressed in V-ASC than in Sc-ASC [8]; [36]–[38]. In addition, these differences in gene expression were apparently independent of gender (data not shown), in spite of potential variations in the local microenvironment due to changes in sex steroids, metabolites, and possibly inflammatory cytokine levels. Thus, differences in gene expression appear to be intrinsic to each ASC population and to persist during in vitro culture, suggesting that they are cell-autonomous and largely unaffected by other (extrinsic) factors.

The overall cytokine profile of the human ASC populations was found to be comparable to that observed in mesenchymal cells derived from human bone marrow (BM-MSC) [39]; [40] and cord blood (CB-MSC) [41], pointing out that the cytokine secretory properties could be viewed as a common characteristic of mesenchymal stem cells, similarly to cell surface marker expression and differentiation into mesenchymal lineages. However, differences in cytokine levels in the CM from distinct ASC subsets further sustain the intrinsic gene expression diversity of each ASC population, since eight of thirteen detectable cytokines were present to a different extent (Fig. 6). These findings reveal heterogeneity in the secretory ability between Sc-ASC and V-ASC and also among ASC_SVF_, ASC_Bottom_ and ASC_Ceiling_. Changes in cellular mRNA levels and protein levels in the culture medium of the individual cytokines were not always coordinated (Figs. 5 and 6), suggesting independent regulation of gene expression and protein secretion, respectively, as previously shown for adiponectin production by Sc and V adipocyte populations [3]. Furthermore, the protein abundance of MCP-1, IL-8, IL-6, and VEGF in the CM was far higher than that of Eotaxin, IL-1ra, FGFbasic and G-CSF (i.e., in the range of 100–600 pg/ml vs. that of 1–50 pg/ml, respectively; Fig. 6), highlighting that the former set of ASC-released cytokines would more likely contribute to the overall adipose tissue cytokine output and to their circulating levels [16]. Noteworthy, ASC_Ceiling_ from the Sc fat depot displayed higher secretion of specific cytokines (i.e., MCP-1, eotaxin, IL-8, IL-6) that regulate leukocyte recruitment and other immune responses, as compared to other Sc-ASC subsets; this feature was not observed in the ASC_Ceiling_ from the V fat depot, suggesting a potentially unique role for this Sc-ASC population in the regulation of inflammatory pathways.

### Conclusions

In conclusion, we have analyzed the expression profiles of distinct ASC isolated from human Sc and V adipose tissue and revealed, for the first time, significant differences in the gene expression profile and related functional networks and in the cytokine release ability according to the anatomical origin of these cells. In addition, the results including the bioinformatics analysis have allowed us to identify ASC_Ceiling_ and ASC_Bottom_ as two ASC populations in addition to the ASC_SVF_ that are characterized by an unmatched gene expression pattern. The knowledge obtained in this study provides further evidence of the heterogeneity and complexity of ASCs existing in abdominal Sc and V adipose tissue in humans.

## Supporting Information

Figure S1 **Adipogenic, osteogenic, and chondrogenic potential of human ASC populations.** ASC_SVF_, ASC_Bottom_, and ASC_Ceiling_ were cultured in adipogenic, osteogenic, or chondrogenic induction media. Histochemical stainings were performed as described under Materials and Methods. Cultures are displayed at 20× magnification (representative of n = 4). *A*. Adipogenic differentiation. *B*. Osteogenic differentiation. *C*. Chondrogenic differentiation.
(TIF)

Figure S2 **Expression of adipose tissue-specific genes in Sc and V ASC differentiated into mature adipocytes.** Sc-ASC (white bars) and V-ASC (black bars) were differentiated as described under Materials and Methods. Total RNA was extracted from undifferentiated ASC and mature adipocytes, respectively, and mRNA expression levels of PPARγ, adiponectin, and GLUT4 were determined by *q*RT-PCR. The mRNA level was normalized for each target gene against 18S ribosomal RNA as internal control. Values are means±SE of cells from five independent donors performed in triplicate and expressed as fold-increase vs. undifferentiated ASC (*p*<0.001). Paired Sc-ASCs and V-ASCs were obtained from 5 non-obese individuals with normal glucose tolerance (3 men, 2 women; age 66±11 yrs; BMI 26.0±2.0 kg/m^2^; fasting plasma glucose 79±9 mg/dl).
(TIF)

Figure S3 **Microarray workflow for whole ASC mRNA transcript analysis.** *A*. Clinical and metabolic parameters of the donors enrolled for the microarray analysis. *B*. The three distinct ASC populations were cultured as described under Materials and Methods. Equal amounts of RNA were isolated from ASC_SVF_, ASC_Bottom_, and ASC_Ceiling_ obtained from Sc and V adipose tissue depots of the donors, and hybridized to Affymetrix chips.
(TIF)

Figure S4 **Hierarchical clusters analysis of Sc and V ASC.** *A.* Hierarchical cluster analysis (HCL) was conducted by PartekGS software including all genes. Groupings showing differential expression were selected and inspected for downstream analyses. Hierarchical clusters analysis of Sc- and V-ASC subsets. *B*. Hierarchical clusters analysis of ASC_SVF_, ASC_Bottom_, and ASC_Ceiling_ from Sc and V fat depots, respectively. Profiles of the transcripts were organized by hierarchical clustering. Genes were analyzed further as described in the text.
(TIF)

Table S1 **Primers used for** ***q*** **RT-PCR.**
(DOCX)

Table S2 **Threshold cycles (Ct) of** ***q*** **RT-PCR for markers expressed by ASCs.** Cells were harvested at passage 4 and RNA was analyzed for ASC markers (CD105, CD44, CD49d), human leukocytes and macrophages (CD45, CD11b), and mature endothelial cells (CD31).
(DOCX)

Table S3 **List of conjoint differentially expressed genes among ASC_SVF_, ASC_Bottom_ and ASC_Ceiling_ from Sc and V adipose tissue.**
(DOCX)

Table S4 **Quantitative array data for the selected genes assessed by** ***q*** **RT-PCR.**
(DOCX)
