# Supplementary figures and images for: Differences in Gene Expression and Cytokine Release Profiles Highlight the Heterogeneity of Distinct Subsets of Adipose Tissue-Derived Stem Cells in the Subcutaneous and Visceral Adipose Tissue in Humans
Source: PLoS One. 2013 Mar 5;8(3):e57892. doi: 10.1371/journal.pone.0057892 (PMC3589487; doi:10.1371/journal.pone.0057892)

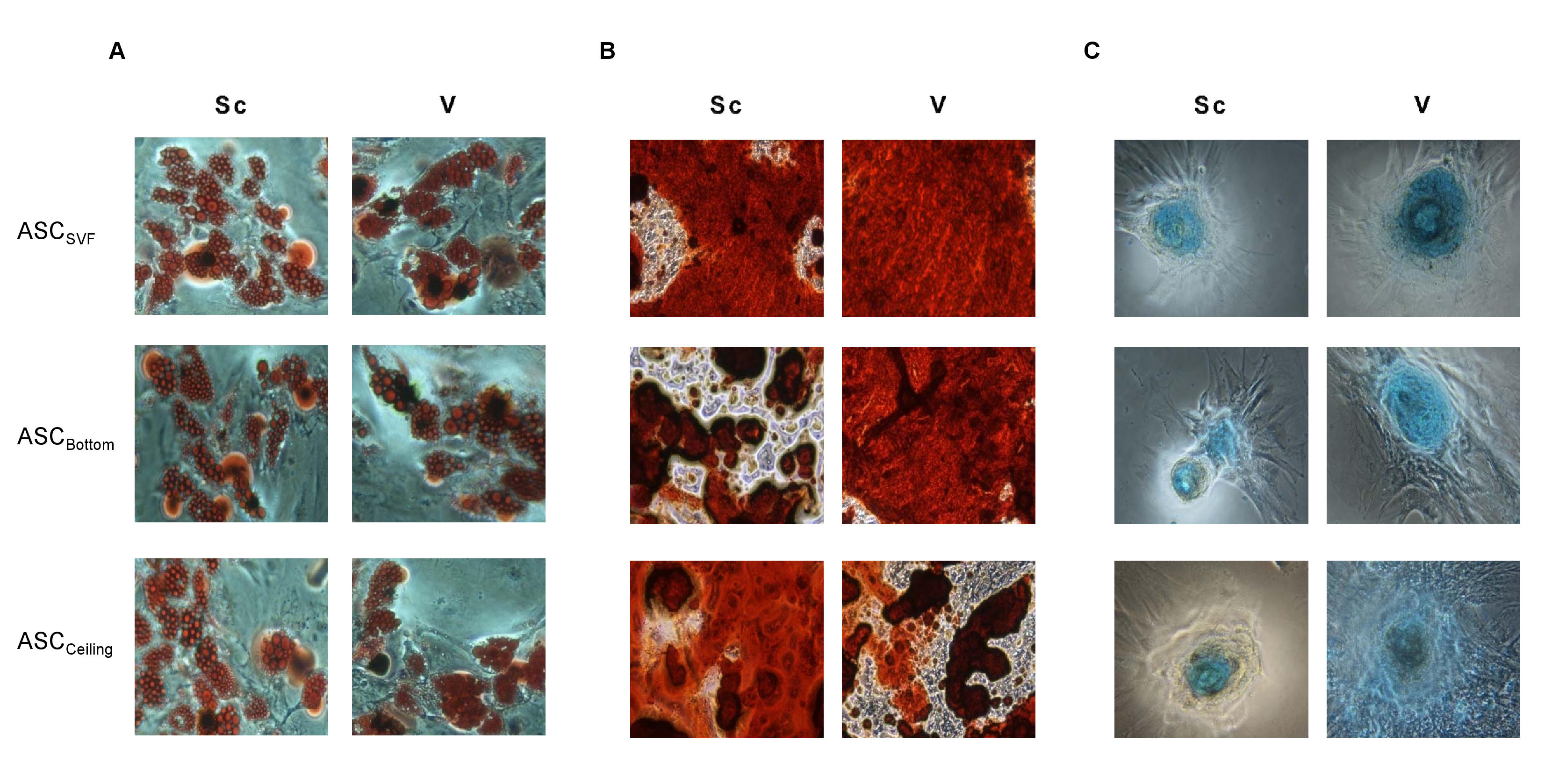

Supplement: Figure S1 — Adipogenic, osteogenic, and chondrogenic potential of human ASC populations. ASCSVF, ASCBottom, and ASCCeiling were cultured in adipogenic, osteogenic, or chondrogenic induction media. Histochemical stainings were performed as described under Materials and Methods. Cultures are displayed at 20× magnification (representative of n = 4). A. Adipogenic differentiation. B. Osteogenic differentiation. C. Chondrogenic differentiation. (TIF) [file pone.0057892.s001.tif]

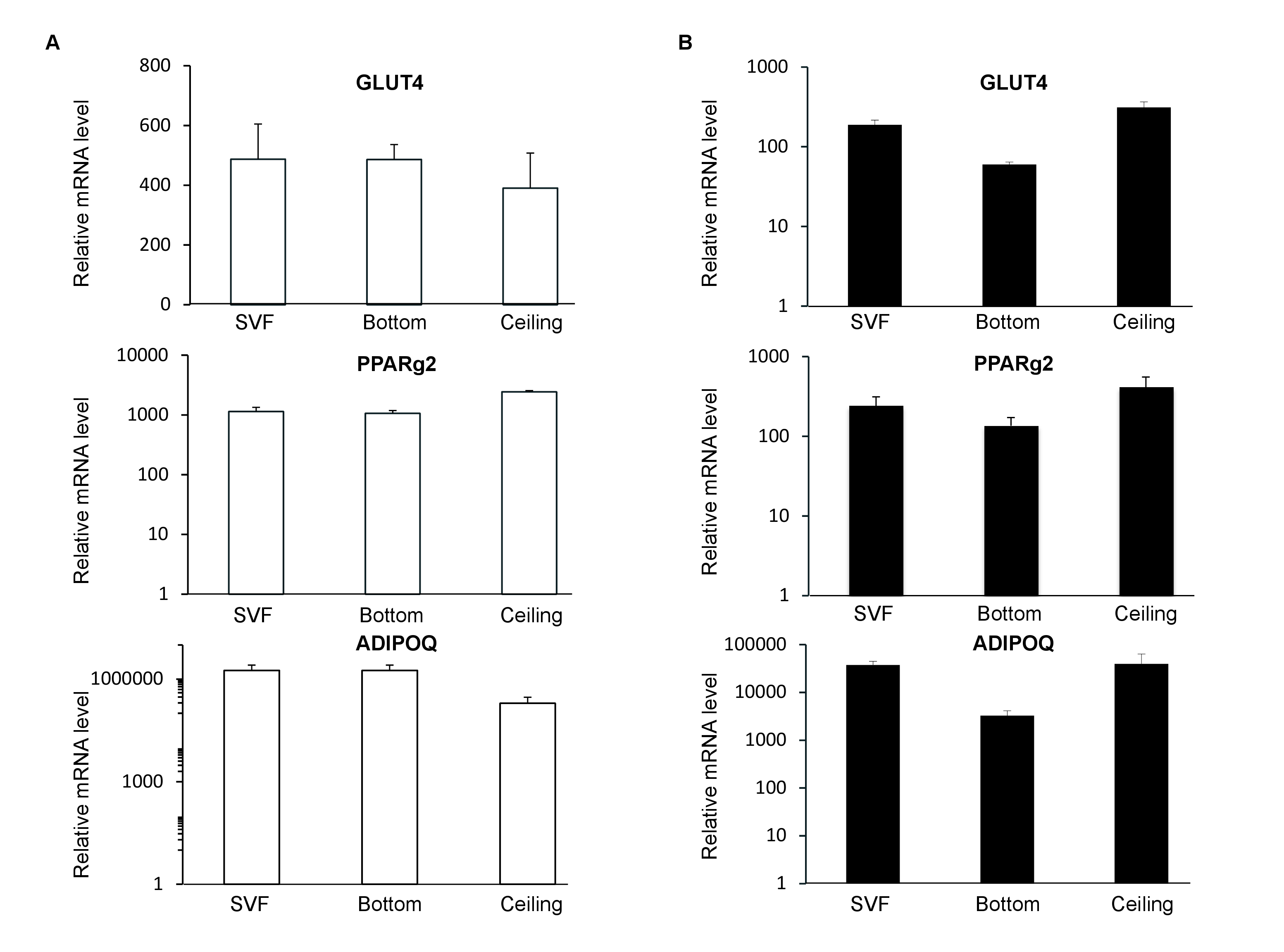

Supplement: Figure S2 — Expression of adipose tissue-specific genes in Sc and V ASC differentiated into mature adipocytes. Sc-ASC (white bars) and V-ASC (black bars) were differentiated as described under Materials and Methods. Total RNA was extracted from undifferentiated ASC and mature adipocytes, respectively, and mRNA expression levels of PPARγ, adiponectin, and GLUT4 were determined by qRT-PCR. The mRNA level was normalized for each target gene against 18S ribosomal RNA as internal control. Values are means±SE of cells from five independent donors performed in triplicate and expressed as fold-increase vs. undifferentiated ASC (p<0.001). Paired Sc-ASCs and V-ASCs were obtained from 5 non-obese individuals with normal glucose tolerance (3 men, 2 women; age 66±11 yrs; BMI 26.0±2.0 kg/m2; fasting plasma glucose 79±9 mg/dl). (TIF) [file pone.0057892.s002.tif]

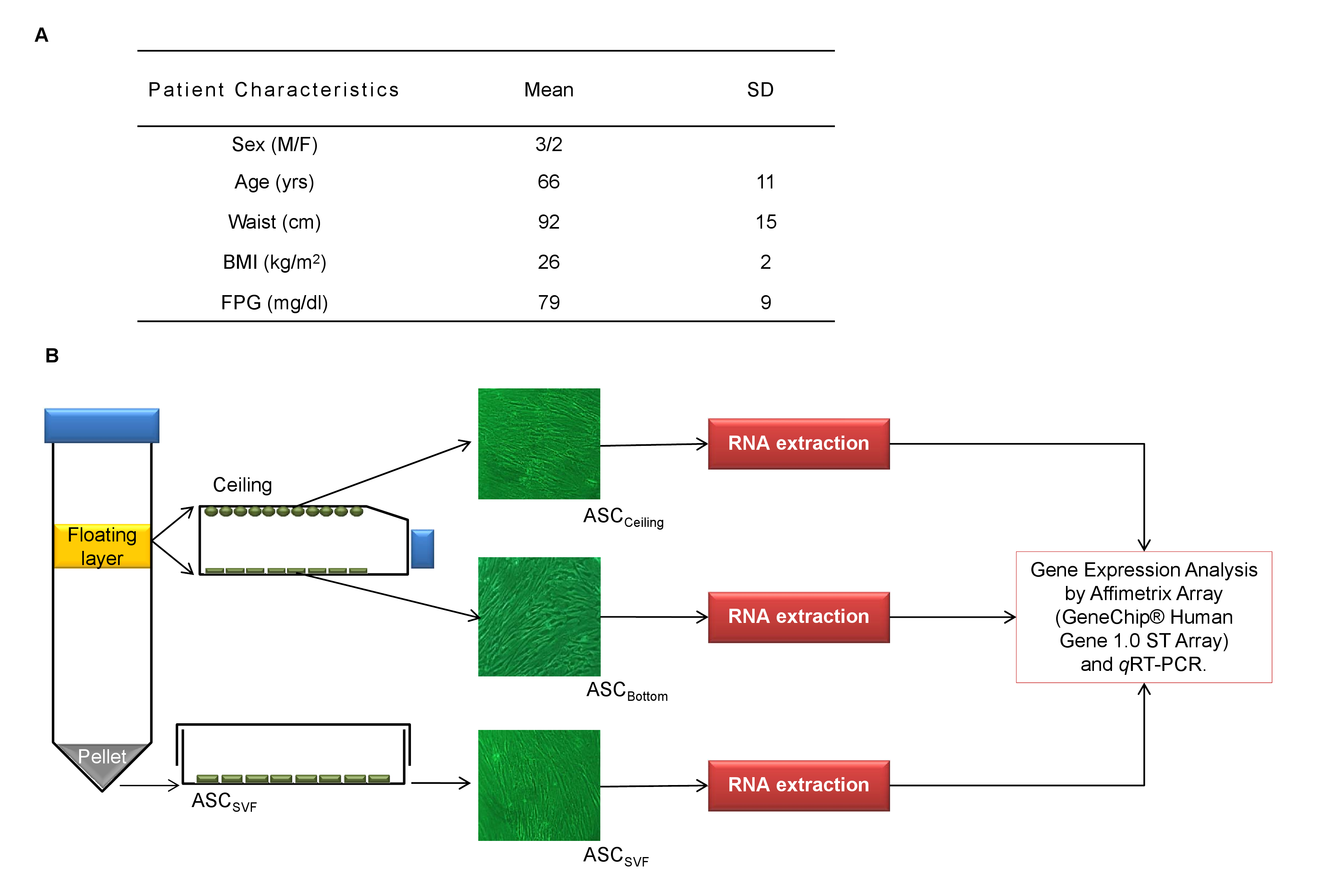

Supplement: Figure S3 — Microarray workflow for whole ASC mRNA transcript analysis. A. Clinical and metabolic parameters of the donors enrolled for the microarray analysis. B. The three distinct ASC populations were cultured as described under Materials and Methods. Equal amounts of RNA were isolated from ASCSVF, ASCBottom, and ASCCeiling obtained from Sc and V adipose tissue depots of the donors, and hybridized to Affymetrix chips. (TIF) [file pone.0057892.s003.tif]

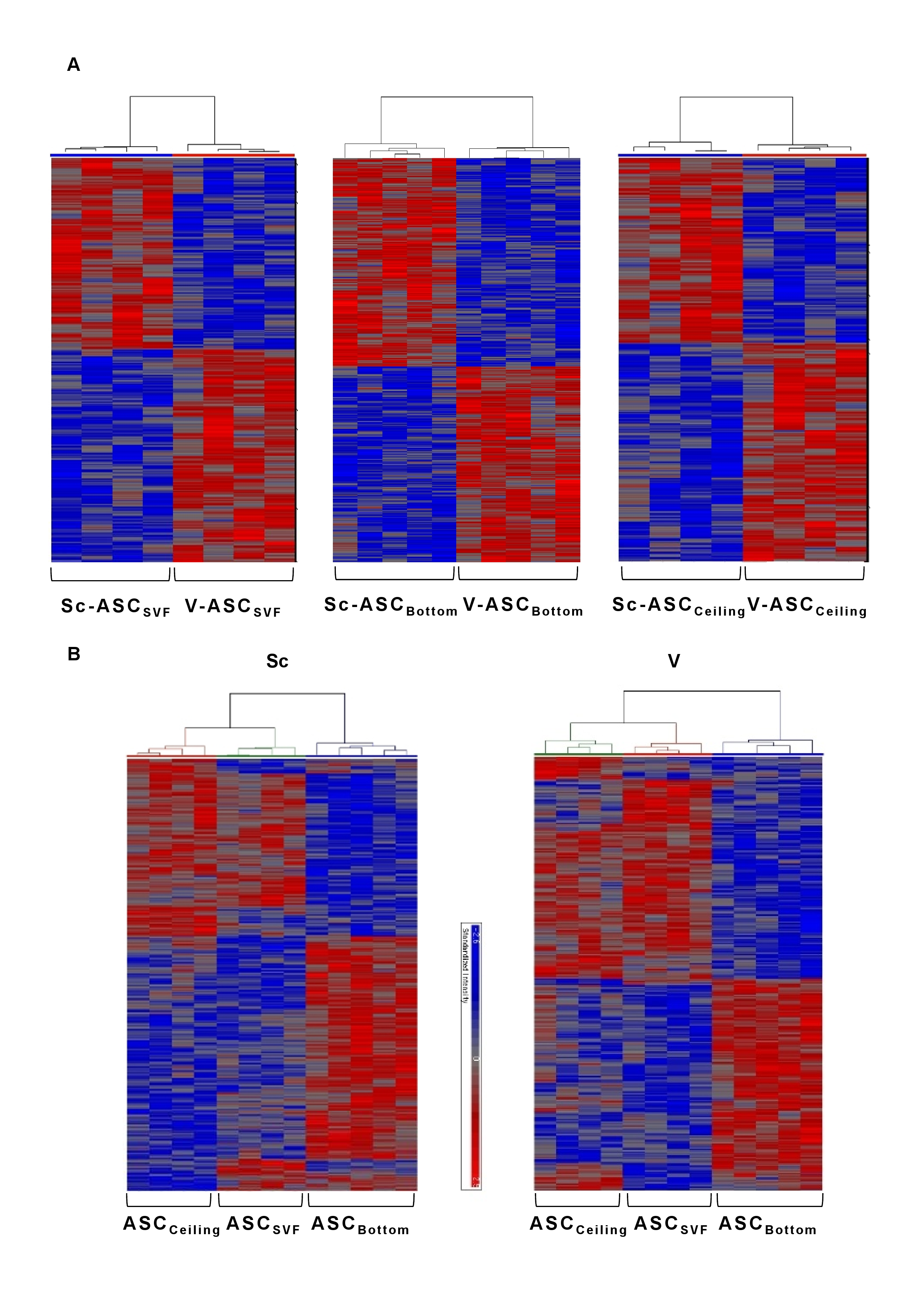

Supplement: Figure S4 — Hierarchical clusters analysis of Sc and V ASC. A. Hierarchical cluster analysis (HCL) was conducted by PartekGS software including all genes. Groupings showing differential expression were selected and inspected for downstream analyses. Hierarchical clusters analysis of Sc- and V-ASC subsets. B. Hierarchical clusters analysis of ASCSVF, ASCBottom, and ASCCeiling from Sc and V fat depots, respectively. Profiles of the transcripts were organized by hierarchical clustering. Genes were analyzed further as described in the text. (TIF) [file pone.0057892.s004.tif]
