# Supplementary material for: Differences in Gene Expression and Cytokine Release Profiles Highlight the Heterogeneity of Distinct Subsets of Adipose Tissue-Derived Stem Cells in the Subcutaneous and Visceral Adipose Tissue in Humans
Source: PLoS One. 2013 Mar 5;8(3):e57892. doi: 10.1371/journal.pone.0057892 (PMC3589487; doi:10.1371/journal.pone.0057892)
Supplement: Table S1 — Primers used for q RT-PCR. (DOCX) [file pone.0057892.s005.docx]

**Table S1.**

| **Target** | **Forward Primer** | **Reverse Primer** |
| --- | --- | --- |
| **18s rRNA** | CGAACGTCTGCCCTATCAACTT | ACCCGTGGTCACCATGGTA |
| **CD105** | TCATCACCACAGCGGAAAAA | GGTAGAGGCCCAGCTGGAA |
| **CD44** | GGATGGATATGGACTCCAGTCATA | TGCTGCGTTGTCATTGAAAGA |
| **CD49d** | ACATCAAGCATTTATGCGGAAA | ACATCAAGCATTTATGCGGAAA |
| **CD106** | GTCAATGTTGCCCCCAGAGA | GTCATATTCACAGAACTGCCTTCCT |
| **CD45** | CACATTCGAGCAATATCAATTCCTA | GATGGTTGTTTTTCTTTACTTGTCC |
| **CD31** | GGAGTCCAGCCGCATATCC | GCTTGGAAAATAGTTCTGTTATGTT |
| **CD11b** | GGCGGATGAAGGAGTTTGTCT | TGCATCAAAGAGAACAAGGTTTTG |
| **HoxA5** | CCGCCCAACCCCAGAT | CCGCCTATGTTGTCATGACTTATG |
| **Tbx15** | AGGACATATCATTCTGCACTCTATG | AACAGGCTTAGTGGGTGAAAGGT |
| **IL-6** | GGTACATCCTCGACGGCATCT | GTGCCTCTTTGCTGCTTTCAC |
| **IL-8** | CCTTGGCAAAACTGCACCTT | CCTTGGCAAAACTGCACCTT |
| **VEGF** | TCATCACGAAGTGGTGAAGTTCA | TCAGGGTACTCCTGGAAGATGTC |
| **MCP-1** | TCGCTCAGCCAGATGCAA | CCTCTGCACTGAGATCTTCCTATTG |
| **PIPTNC1** | CGTGGAGAGAGAAGTTTGCTTTATT | TGCTTAGGATCCTCAGATTCTTTGT |
| **FABP5** | CCCTGGGAGAGAAGTTTGAAGA | AATGCACCATCTGTAAAGTTGCA |
| **PI16** | CTCTGGCTACCACTGCAAGAACT | CTCTGGCTACCACTGCAAGAACT |
| **TFPI2** | CAACAGGAAATAACGCGGAGAT | TCTGCGTGTACCTGTCGTAGTAGTA |
| **ANXA10** | TCCAAGGATTTGACTGTGACAAA | GGTATGCCTCTGCAATCATCATC |
| **MMP3** | ACAAAGGATACAACAGGGACCAA | TAGAGTGGGTACATCAAAGCTTCA |
