# Supplementary material for: Differences in Gene Expression and Cytokine Release Profiles Highlight the Heterogeneity of Distinct Subsets of Adipose Tissue-Derived Stem Cells in the Subcutaneous and Visceral Adipose Tissue in Humans
Source: PLoS One. 2013 Mar 5;8(3):e57892. doi: 10.1371/journal.pone.0057892 (PMC3589487; doi:10.1371/journal.pone.0057892)
Supplement: Table S2 — Threshold cycles (Ct) of q RT-PCR for markers expressed by ASCs. Cells were harvested at passage 4 and RNA was analyzed for ASC markers (CD105, CD44, CD49d), human leukocytes and macrophages (CD45, CD11b), and mature endothelial cells (CD31). (DOCX) [file pone.0057892.s006.docx]

**Table S2.**

| Marker | Sc-ASC | | | V-ASC | | |
| --- | --- | --- | --- | --- | --- | --- |
|  | SVF | Bottom | Ceiling | SVF | Bottom | Ceiling |
| CD105 | 17.8 | 19.9 | 19.5 | 17 | 19.2 | 19.9 |
| CD44 | 25.8 | 26.1 | 23.8 | 25.7 | 26 | 26.8 |
| CD49d | 24.9 | 26 | 29.9 | 25.9 | 26.7 | 27.1 |
| CD106  (VCAM-I) | >30 | >30 | >30 | >30 | >30 | >30 |
| CD45  (LCA) | >30 | >30 | >30 | >30 | >30 | >30 |
| CD31 | >30 | >30 | >30 | >30 | >30 | >30 |
| CD11b | >30 | >30 | >30 | >30 | >30 | >30 |
