# Supplementary material for: Differences in Gene Expression and Cytokine Release Profiles Highlight the Heterogeneity of Distinct Subsets of Adipose Tissue-Derived Stem Cells in the Subcutaneous and Visceral Adipose Tissue in Humans
Source: PLoS One. 2013 Mar 5;8(3):e57892. doi: 10.1371/journal.pone.0057892 (PMC3589487; doi:10.1371/journal.pone.0057892)
Supplement: Table S3 — List of conjoint differentially expressed genes among ASCSVF, ASCBottom and ASCCeiling from Sc and V adipose tissue. (DOCX) [file pone.0057892.s007.docx]

**Table S3.**

| Reference Sequence and Gene Symbol | p-value  (V SVF vs. V Bottom) | Fold-Change  (V SVF vs. V Bottom) | p-value  (V SVF vs. V Ceiling) | Fold-Change  (V SVF vs. V Ceiling) | p-value  (V Bottom vs. V Ceiling) | Fold-Change  (V Bottom vs. V Ceiling) |
| --- | --- | --- | --- | --- | --- | --- |
| NM_002977 SCN9A | 0.1 | -1.59 | 0.03 | -2.49 | 0.23 | -1.59565 |
| NM_007029 STMN2 | 0.08 | -1.61 | 0.03 | -7.6 | 0.07 | -4.72 |
| Unknown | 0.00 | -2.93 | 0.05 | -1.5 | 0.00 | 1.86 |
| NM_006528  TFPI2 | 0.02 | -2.28 | 0.00 | -4.82 | 0.13 | -2.11 |
|  | | | | | | |
| Reference Sequence and Gene Symbol | p-value  (Sc SVF vs. V SVF) | Fold-Change  (Sc SVF vs. V SVF) | p-value  (Sc Bottom vs. V Bottom) | Fold-Change  (Sc Bottom vs. V Bottom) | p-value  (Sc Ceiling vs. V Ceiling) | Fold-Change  (Sc Ceiling vs. V Ceiling) |
| NM_005584 MAB21L1 | 9.12E-05 | 5.11337 | 0.00 | 4.49 | 0.00 | 2.97 |
