# Supplementary material for: Differences in Gene Expression and Cytokine Release Profiles Highlight the Heterogeneity of Distinct Subsets of Adipose Tissue-Derived Stem Cells in the Subcutaneous and Visceral Adipose Tissue in Humans
Source: PLoS One. 2013 Mar 5;8(3):e57892. doi: 10.1371/journal.pone.0057892 (PMC3589487; doi:10.1371/journal.pone.0057892)
Supplement: Table S4 — Quantitative array data for the selected genes assessed by q RT-PCR. (DOCX) [file pone.0057892.s008.docx]

**Table S4.**

| **Source of ASC** | **Gene** | **p-value**  **(SVF vs. Bottom)** | **Fold-change**  **(SVF vs. Bottom)** | **p-value**  **(SVF vs. Ceiling)** | **Fold-change**  **(SVF vs. Ceiling)** | **p-value**  **(Bottom vs. Ceiling)** | **Fold-change**  **(Bottom vs. Ceiling)** |
| --- | --- | --- | --- | --- | --- | --- | --- |
| Sc-ASC | HOXA5 | 0.22 | 1.39 | 0.44 | 1.20 | 0.66 | 0.86 |
| V-ASC | HOXA5 | 0.07 | 1.55 | 0.85 | -1.07 | 0.06 | 1.76 |
| Sc-ASC | TBX15 | 0.99 | 1.00 | 0.93 | 1.01 | 0.94 | 1.01 |
| V-ASC | TBX15 | 0.33 | 1.12 | 0.08 | -1.79 | 0.04 | -1.40 |
| Sc-ASC | IL-6 | 0.95 | 0.97 | 0.02 | -2.16 | 0.63 | -2.10 |
| V-ASC | IL-6 | 0.97 | -1.02 | 0.82 | -1.21 | 0.76 | -1.18 |
| Sc-ASC | IL-8 | 0.40 | 0.61 | 0.04 | -3.65 | 0.15 | -2.24 |
| V-ASC | IL-8 | 0.74 | 1.08 | 0.19 | -2.22 | 0.17 | -2.05 |
| Sc-ASC | VEGF | 0.56 | -1.12 | 0.05 | -1.53 | 0.20 | -1.4 |
| V-ASC | VEGF | 0.70 | -1.11 | 0.42 | -1.19 | 0.06 | -1.66 |
| Sc-ASC | MCP-1 | 0.63 | -1.18 | 0.31 | -1.26 | 0.86 | -1.06 |
| V-ASC | MCP-1 | 0.88 | -1.03 | 0.37 | -1.45 | 0.32 | -1.71 |
| Sc-ASC | PITPNC1 |  |  |  |  | 0.00 | -1.72 |
| Sc-ASC | FABP5 |  |  | 0.03 | -2.26 |  |  |
| Sc-ASC | PI16 |  |  | 0.00 | -7.32 |  |  |
| V-ASC | TFP12 |  |  |  |  | 0.18 | -2.11 |
| V-ASC | ANXA10 |  |  | 0.05 | -3.28 |  |  |
| V-ASC | MMP3 |  |  | 0.03 | -3.07 |  |  |
